# Redetermined crystal structure of *N*-(β-carb­oxy­eth­yl)-α-isoleucine

**DOI:** 10.1107/S2056989015014498

**Published:** 2015-08-22

**Authors:** M. Chandrarekha, N. Srinivasan, R. V. Krishnakumar

**Affiliations:** a Department of Physics, Thiagarajar College, Madurai 625 009, Tamil Nadu, India

**Keywords:** crystal structure, amino acids, ionization state, hydrogen bonding, isoleucine

## Abstract

Redetermination of the crystal structure of *N*-(β-carb­oxy­eth­yl)-α-isoleucine, C_9_H_18_N_2_O_3_, reported earlier by Nehls *et al.* [*Acta Cryst.* (2013), E**69**, o172–o173], was undertaken in which the ionization state assigned to the mol­ecule as unionized has been modified as zwitterionic in the present work. Single-crystal X-ray intensity data obtained from freshly grown crystals and freely refining the amino H atoms provide enhanced refinement and structural parameters, particularly the hydrogen-bonding scheme. N—H⋯O hydrogen bonds dominate the inter­molecular inter­actions along with a C—H⋯O hydrogen bond. The inter­molecular inter­action pattern is a three-dimensional network. The structure was refined as a two-component perfect inversion twin.

## Related literature   

For earlier work on the crystal structure of *N*-(β-carb­oxy­eth­yl)-α-isoleucine, see: Nehls *et al.* (2013 ▸). For the crystal structure of l-isoleucine and its indolylacetyl derivative, respectively, see Görbitz & Dalhus (1996 ▸); Kojić-Prodić *et al.* (1991 ▸). For the importance of freely refining the positions of amino-group H atoms, see: Görbitz (2014 ▸). For absolute configuration and structure parameters, see Flack (1983 ▸); Flack & Bernardinelli (2000 ▸); Hooft *et al.* (2008 ▸); Spek (2009 ▸); Parsons *et al.* (2013 ▸). For chiral and achiral crystal structures, see Flack (2003 ▸). 
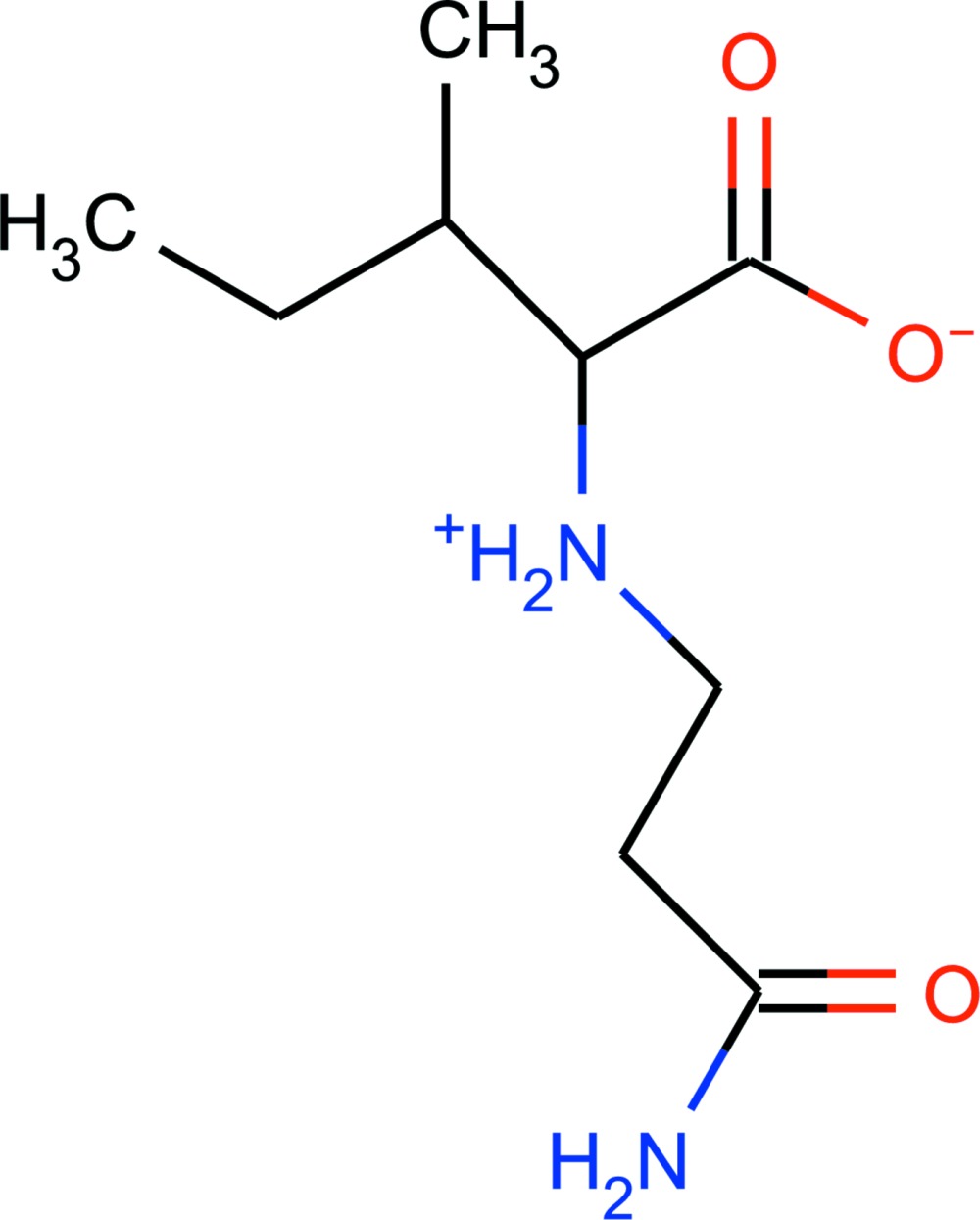


## Experimental   

### Crystal data  

C_9_H_18_N_2_O_3_
*M* *_r_* = 202.25
Orthorhombic, 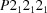

*a* = 5.2996 (5) Å
*b* = 9.0053 (7) Å
*c* = 23.211 (2) Å
*V* = 1107.75 (17) Å^3^
*Z* = 4
Mo *K*α radiation
μ = 0.09 mm^−1^
*T* = 293 K
0.26 × 0.18 × 0.10 mm

### Data collection  

Bruker APEXII CCD diffractometer
Absorption correction: multi-scan (*SADABS*; Bruker, 2009 ▸) *T*_min_ = 0.97, *T*_max_ = 0.99
22904 measured reflections
3127 independent reflections
2538 reflections with *I* > 2σ(*I*)
*R*_int_ = 0.036

### Refinement  

*R*[*F* ^2^ > 2σ(*F* ^2^)] = 0.042
*wR*(*F* ^2^) = 0.102
*S* = 1.08
3127 reflections
146 parameters
H atoms treated by a mixture of independent and constrained refinement
Δρ_max_ = 0.30 e Å^−3^
Δρ_min_ = −0.19 e Å^−3^
Absolute structure: refined as a perfect inversion twin.
Absolute structure parameter: fixed at 0.5 and not refined

### 

Data collection: *APEX2* (Bruker, 2009 ▸); cell refinement: *SAINT* (Bruker, 2009 ▸); data reduction: *SAINT*; program(s) used to solve structure: *SHELXS2013* (Sheldrick, 2008 ▸); program(s) used to refine structure: *SHELXL2014* (Sheldrick, 2015 ▸); molecular graphics: *PLATON* (Spek, 2009 ▸); software used to prepare material for publication: *SHELXL2014*.

## Supplementary Material

Crystal structure: contains datablock(s) I, New_Global_Publ_Block. DOI: 10.1107/S2056989015014498/bg2563sup1.cif

Structure factors: contains datablock(s) I. DOI: 10.1107/S2056989015014498/bg2563Isup2.hkl

Supporting information file. DOI: 10.1107/S2056989015014498/bg2563Isup3.cml

**Fig. 1.**
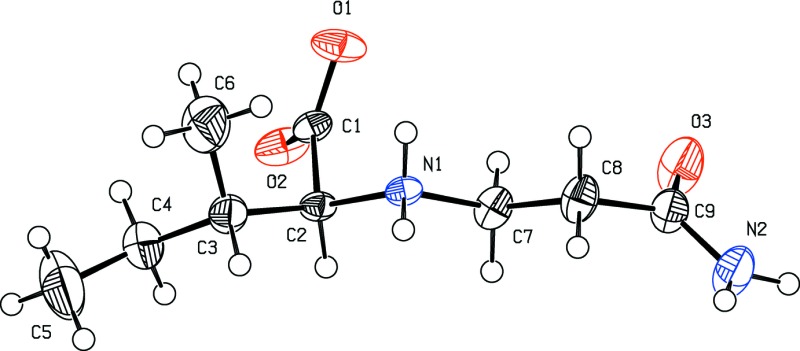
Thermal ellipsoid plot of the title compound, showing the atom numbering scheme.

. DOI: 10.1107/S2056989015014498/bg2563fig1.tif
Thermal ellipsoid plot of the title compound, showing the atom numbering scheme.

**Fig. 2.**
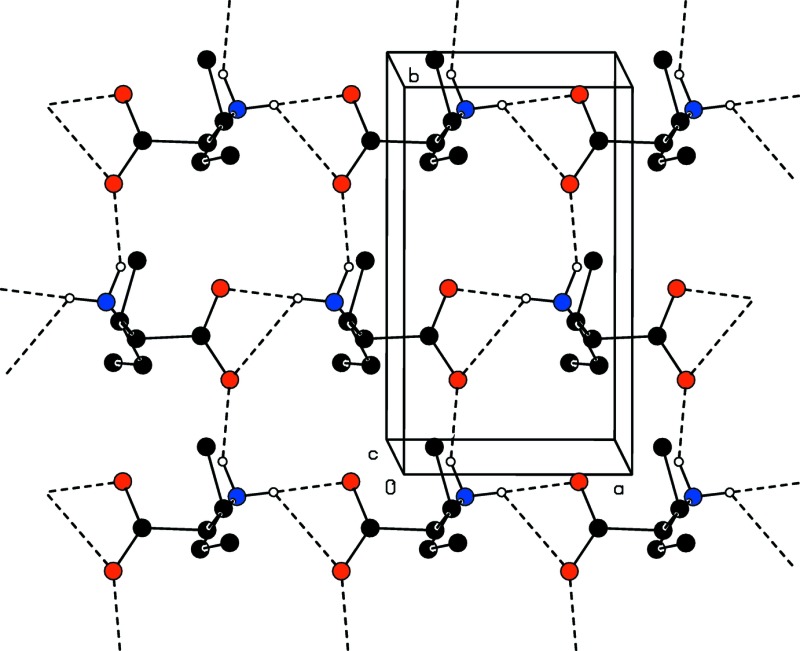
The characteristic head-to-tail N—H···O hydrogen bonds involving the carboxylate and the amino groups. Non pariticipating N-carboxyethl group atoms have been omitted for clarity.

**Fig. 3.**
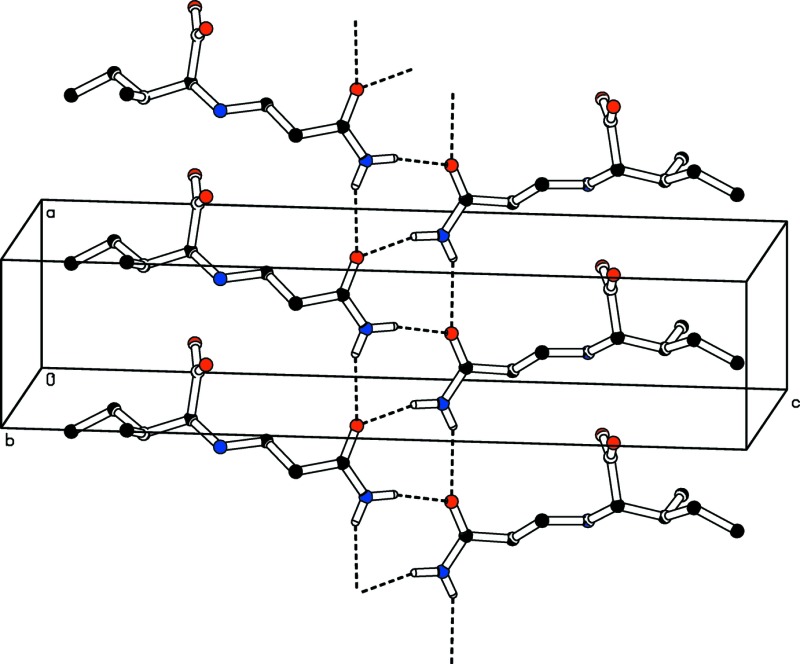
Carbamoyl group N2 and O3 forming N—H···O hydrogen-bonds within themselves leading to C32(8) chains linking screw related molecules along the a axis.

N . DOI: 10.1107/S2056989015014498/bg2563fig2.tif
The characteristic head-to-tail N—H⋯O hydrogen bonds involving the carboxyl­ate and the amino groups. Non pariticipating *N*-carb­oxy­ethl group atoms have been omitted for clarity.

a . DOI: 10.1107/S2056989015014498/bg2563fig3.tif
Carbamoyl group N2 and O3 forming N—H⋯O hydrogen-bonds within themselves leading to C32(8) chains linking screw related mol­ecules along the *a* axis.

CCDC reference: 1416394

Additional supporting information:  crystallographic information; 3D view; checkCIF report

## Figures and Tables

**Table 1:** Hydrogen-bond geometry (, )

*D*H*A*	*D*H	H*A*	*D* *A*	*D*H*A*
N1H1*N*1O2^i^	0.89(2)	1.96(2)	2.7772(19)	152(2)
N1H2*N*1O1^ii^	0.88(2)	1.83(2)	2.7047(19)	174(2)
N2H1*N*2O3^iii^	0.90(3)	2.11(3)	2.970(3)	161(3)
N2H2*N*2O3^ii^	0.84(3)	2.34(3)	3.092(3)	149(2)
C2H2O1^iv^	0.98	2.53	3.469(2)	160

Symmetry codes: (i) 
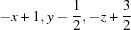
; (ii) 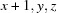; (iii) 
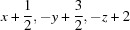
; (iv) 
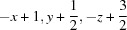
.

## supplementary crystallographic information

### S1. Introduction

Amino acids in their free form exist as 'zwitterions' in their crystals with a deprotonated carboxyl group (COO–) and a protonated NH_3_^+^ group (NH_2_^+^ in proline). Any deviation from this general preferences of amino acids is worth careful considerations. The motivation for the present work is the unionized state reported by Nehls *et al.*, 2013, for the title compound in contrast to the usually preferred 'zwitterionic' state. In this context, redetermination of the crystal structure of the title compouned was undertaken.

### S2.1. Synthesis and crystallization

For crystallization details, see Nehls *et al.* (2013).

### S2.2. Refinement

Coordinates were refined for amino H atoms; other H atoms were positioned with idealized geometry, with fixed C— H = 0.98 (methyl), 0.99 (methyl­ene) or 1.00 Å (methine). U_iso_(H) values were set at 1.2U_eq_ of the carrier atom or at 1.5U_eq_ for methyl and amino groups. The absolute configuration could not be determined by anomalous-dispersion effects in the X-ray diffraction measurements of the crystal, but assigned as L- based on an unchanged chiral centre in the synthetic procedure. The absoulte structure was refined as a perfect inversion twin.

### S3. Results and discussion

Nehls *et al.*, (2013) seem to have presumed an unionized state for the title compound with an undissociated carboxyl (COOH) and a deprotonated amino (NH) group. A scrutiny of the work by Nehls *et al.* revealed that all the H-atoms, including the donor group H atoms were assigned an idealized geometry and refined as riding on their respective non-H atoms to which they are attached. Redetermination of the crystal structure carried out by measuring X-ray intensity data from freshly grown crystals and freely refining the amino-H atoms clearly indicate that the title compound indeed exist as a zwitterion. The correct assignment of the ionized state provided enhanced refinement and structural parameters. Thus, the present redtermination demonstrates the importance of freely refining donor group hydrogens. The S,S (equivalently L-) absolute configuration is deduced from the synthetic pathway as the starting material involved L-isoleucine. The absoulte structure was refined as a perfect inversion twin in order that the Flack x (Flack, 1983; Flack & Bernardinelli, 2000; Parsons *et al.*, 2013) and Hooft y parameters (Spek, 2009) showed good agreement.

The correct assignment of the ionization state to the title compound as 'zwitterion' presents an acceptable description of the inter­molecular inter­action patterns with all the amino-H atoms participating in them. The carboxyl­ate O1 atom of the amino acid derivative participates in a strong head-to-tail N—H···O hydrogen bond characteristic of amino acids, in addition to a C—H···O hydrogen-bond as acceptor. This has consequently resulted in the lengthening of the C6—O1[1.253 (2)Å] bond compared to its counterpart C6—O2[1.234 (2)Å]. The carbamoyl group N2 and O3 form N—H···O hydrogen-bonds within themselves leading to C^3^_2_(8) chains linking screw related molecules along the shortest a-axis. The respective amino and carboxyl­ate group N and O atoms form characteristic head-to-tail hydrogen-bonds leading to a layers parallel to the ab-plane. The inter­molecular inter­action pattern is a three-dimensional network dominated by N—H···O hydrogen bonds, in addition to a C—H···O hydrogen-bond involving the α-carbon atom (C2) as donor and the carboxyl­ate O1 as acceptor.

### Crystal data

**Table:**

C_9_H_18_N_2_O_3_	*F*(000) = 440
*M**_r_* = 202.25	*D*_x_ = 1.213 Mg m^−^^3^*D*_m_ = 1.21 Mg m^−^^3^*D*_m_ measured by floatation method
Orthorhombic, *P*2_1_2_1_2_1_	Mo *K*α radiation, λ = 0.71073 Å
*a* = 5.2996 (5) Å	µ = 0.09 mm^−^^1^
*b* = 9.0053 (7) Å	*T* = 293 K
*c* = 23.211 (2) Å	Needle, colourless
*V* = 1107.75 (17) Å^3^	0.26 × 0.18 × 0.10 mm
*Z* = 4	

### Data collection

**Table:**

Bruker APEXII CCD diffractometer	2538 reflections with *I* > 2σ(*I*)
ω and φ scans	*R*_int_ = 0.036
Absorption correction: multi-scan (*SADABS*; Bruker, 2009)	θ_max_ = 29.9°, θ_min_ = 2.4°
*T*_min_ = 0.97, *T*_max_ = 0.99	*h* = −7→7
22904 measured reflections	*k* = −12→11
3127 independent reflections	*l* = −31→32

### Refinement

**Table:**

Refinement on *F*^2^	Hydrogen site location: mixed
Least-squares matrix: full	H atoms treated by a mixture of independent and constrained refinement
*R*[*F*^2^ > 2σ(*F*^2^)] = 0.042	*w* = 1/[σ^2^(*F*_o_^2^) + (0.0479*P*)^2^ + 0.1014*P*] where *P* = (*F*_o_^2^ + 2*F*_c_^2^)/3
*wR*(*F*^2^) = 0.102	(Δ/σ)_max_ < 0.001
*S* = 1.08	Δρ_max_ = 0.30 e Å^−^^3^
3127 reflections	Δρ_min_ = −0.19 e Å^−^^3^
146 parameters	Absolute structure: Refined as a perfect inversion twin.
0 restraints	Absolute structure parameter: 0.5

### Special details

**Table:**

Geometry. All e.s.d.'s (except the e.s.d. in the dihedral angle between two l.s. planes) are estimated using the full covariance matrix. The cell e.s.d.'s are taken into account individually in the estimation of e.s.d.'s in distances, angles and torsion angles; correlations between e.s.d.'s in cell parameters are only used when they are defined by crystal symmetry. An approximate (isotropic) treatment of cell e.s.d.'s is used for estimating e.s.d.'s involving l.s. planes.
Refinement. Refined as a 2-component perfect inversion twin.

### Fractional atomic coordinates and isotropic or equivalent isotropic displacement parameters (Å^2^)

**Table:**

	*x*	*y*	*z*	*U*_iso_*/*U*_eq_	
O1	0.2868 (2)	0.54162 (13)	0.74549 (7)	0.0379 (4)	
O2	0.2473 (3)	0.77532 (13)	0.71778 (7)	0.0383 (4)	
O3	0.6665 (3)	0.6614 (2)	0.94137 (7)	0.0550 (5)	
N1	0.7861 (3)	0.57896 (16)	0.76272 (6)	0.0213 (3)	
N2	1.0857 (4)	0.6555 (3)	0.95384 (9)	0.0475 (5)	
C5	0.7705 (8)	0.7167 (5)	0.55526 (12)	0.0942 (12)	
H5A	0.9472	0.7357	0.5606	0.141*	
H5B	0.7017	0.7884	0.5290	0.141*	
H5C	0.7479	0.6188	0.5398	0.141*	
C4	0.6370 (5)	0.7281 (3)	0.61215 (10)	0.0506 (6)	
H4A	0.4584	0.7101	0.6062	0.061*	
H4B	0.6555	0.8285	0.6267	0.061*	
C3	0.7352 (4)	0.6194 (2)	0.65736 (8)	0.0329 (4)	
H3	0.9199	0.6235	0.6558	0.040*	
C6	0.6603 (6)	0.4612 (3)	0.64363 (10)	0.0571 (7)	
H6A	0.7232	0.3961	0.6731	0.086*	
H6B	0.7304	0.4330	0.6071	0.086*	
H6C	0.4797	0.4540	0.6420	0.086*	
C2	0.6583 (3)	0.66957 (19)	0.71782 (7)	0.0224 (4)	
H2	0.7123	0.7729	0.7227	0.027*	
C1	0.3732 (3)	0.66295 (19)	0.72788 (8)	0.0244 (4)	
C7	0.7684 (4)	0.6481 (2)	0.82050 (8)	0.0297 (4)	
H7A	0.5926	0.6536	0.8319	0.036*	
H7B	0.8340	0.7485	0.8187	0.036*	
C8	0.9134 (4)	0.5614 (2)	0.86493 (8)	0.0354 (5)	
H8A	1.0912	0.5611	0.8551	0.042*	
H8B	0.8547	0.4594	0.8655	0.042*	
C9	0.8776 (4)	0.6301 (2)	0.92372 (8)	0.0348 (4)	
H1N1	0.723 (4)	0.488 (2)	0.7648 (9)	0.029 (5)*	
H2N1	0.947 (4)	0.568 (2)	0.7544 (9)	0.027 (5)*	
H2N2	1.228 (6)	0.631 (3)	0.9411 (11)	0.051 (7)*	
H1N2	1.082 (6)	0.697 (3)	0.9890 (13)	0.064 (9)*	

### Atomic displacement parameters (Å^2^)

**Table:**

	*U*^11^	*U*^22^	*U*^33^	*U*^12^	*U*^13^	*U*^23^
O1	0.0150 (6)	0.0337 (7)	0.0651 (10)	−0.0005 (5)	0.0039 (6)	0.0146 (6)
O2	0.0213 (7)	0.0261 (6)	0.0675 (10)	0.0044 (6)	−0.0089 (7)	−0.0012 (6)
O3	0.0324 (8)	0.0925 (14)	0.0400 (9)	0.0071 (9)	0.0034 (7)	−0.0200 (8)
N1	0.0129 (6)	0.0224 (7)	0.0287 (8)	−0.0001 (5)	0.0007 (5)	−0.0012 (6)
N2	0.0339 (11)	0.0737 (15)	0.0348 (10)	−0.0011 (10)	−0.0030 (8)	−0.0149 (10)
C5	0.101 (3)	0.142 (3)	0.0405 (15)	−0.019 (3)	0.0009 (16)	0.0249 (17)
C4	0.0549 (15)	0.0597 (14)	0.0371 (11)	−0.0110 (12)	−0.0076 (11)	0.0114 (11)
C3	0.0225 (9)	0.0464 (11)	0.0299 (9)	−0.0034 (9)	0.0010 (8)	−0.0003 (8)
C6	0.080 (2)	0.0488 (14)	0.0423 (13)	0.0016 (14)	0.0050 (13)	−0.0135 (11)
C2	0.0141 (7)	0.0235 (8)	0.0296 (8)	−0.0012 (6)	−0.0007 (6)	0.0019 (7)
C1	0.0142 (7)	0.0270 (8)	0.0319 (9)	−0.0006 (7)	−0.0014 (7)	−0.0027 (7)
C7	0.0266 (9)	0.0320 (9)	0.0305 (9)	0.0073 (8)	−0.0013 (7)	−0.0079 (7)
C8	0.0320 (11)	0.0428 (11)	0.0313 (10)	0.0088 (9)	−0.0057 (8)	−0.0070 (9)
C9	0.0319 (10)	0.0430 (11)	0.0295 (9)	0.0018 (9)	−0.0019 (9)	−0.0027 (8)

### Geometric parameters (Å, º)

**Table:**

O1—C1	1.253 (2)	C4—H4B	0.9700
O2—C1	1.234 (2)	C3—C6	1.513 (3)
O3—C9	1.224 (3)	C3—C2	1.530 (2)
N1—C7	1.481 (2)	C3—H3	0.9800
N1—C2	1.487 (2)	C6—H6A	0.9600
N1—H1N1	0.89 (2)	C6—H6B	0.9600
N1—H2N1	0.88 (2)	C6—H6C	0.9600
N2—C9	1.326 (3)	C2—C1	1.530 (2)
N2—H2N2	0.84 (3)	C2—H2	0.9800
N2—H1N2	0.90 (3)	C7—C8	1.504 (3)
C5—C4	1.502 (4)	C7—H7A	0.9700
C5—H5A	0.9600	C7—H7B	0.9700
C5—H5B	0.9600	C8—C9	1.510 (3)
C5—H5C	0.9600	C8—H8A	0.9700
C4—C3	1.527 (3)	C8—H8B	0.9700
C4—H4A	0.9700		
			
C7—N1—C2	112.03 (13)	H6A—C6—H6B	109.5
C7—N1—H1N1	108.3 (13)	C3—C6—H6C	109.5
C2—N1—H1N1	111.9 (14)	H6A—C6—H6C	109.5
C7—N1—H2N1	107.9 (14)	H6B—C6—H6C	109.5
C2—N1—H2N1	110.4 (14)	N1—C2—C3	111.07 (14)
H1N1—N1—H2N1	106.1 (19)	N1—C2—C1	108.74 (14)
C9—N2—H2N2	121.0 (18)	C3—C2—C1	113.06 (15)
C9—N2—H1N2	122 (2)	N1—C2—H2	107.9
H2N2—N2—H1N2	117 (3)	C3—C2—H2	107.9
C4—C5—H5A	109.5	C1—C2—H2	107.9
C4—C5—H5B	109.5	O2—C1—O1	125.42 (16)
H5A—C5—H5B	109.5	O2—C1—C2	118.20 (15)
C4—C5—H5C	109.5	O1—C1—C2	116.38 (15)
H5A—C5—H5C	109.5	N1—C7—C8	111.73 (14)
H5B—C5—H5C	109.5	N1—C7—H7A	109.3
C5—C4—C3	113.6 (2)	C8—C7—H7A	109.3
C5—C4—H4A	108.9	N1—C7—H7B	109.3
C3—C4—H4A	108.9	C8—C7—H7B	109.3
C5—C4—H4B	108.9	H7A—C7—H7B	107.9
C3—C4—H4B	108.9	C7—C8—C9	110.04 (16)
H4A—C4—H4B	107.7	C7—C8—H8A	109.7
C6—C3—C4	111.70 (19)	C9—C8—H8A	109.7
C6—C3—C2	113.68 (17)	C7—C8—H8B	109.7
C4—C3—C2	110.48 (17)	C9—C8—H8B	109.7
C6—C3—H3	106.9	H8A—C8—H8B	108.2
C4—C3—H3	106.9	O3—C9—N2	122.98 (19)
C2—C3—H3	106.9	O3—C9—C8	120.75 (18)
C3—C6—H6A	109.5	N2—C9—C8	116.27 (19)
C3—C6—H6B	109.5		
			
C5—C4—C3—C6	−71.2 (3)	N1—C2—C1—O2	144.24 (16)
C5—C4—C3—C2	161.2 (2)	C3—C2—C1—O2	−91.9 (2)
C7—N1—C2—C3	165.30 (14)	N1—C2—C1—O1	−36.3 (2)
C7—N1—C2—C1	−69.67 (18)	C3—C2—C1—O1	87.5 (2)
C6—C3—C2—N1	62.6 (2)	C2—N1—C7—C8	−176.01 (15)
C4—C3—C2—N1	−170.95 (17)	N1—C7—C8—C9	−176.58 (17)
C6—C3—C2—C1	−60.0 (2)	C7—C8—C9—O3	48.8 (3)
C4—C3—C2—C1	66.5 (2)	C7—C8—C9—N2	−130.4 (2)

### Hydrogen-bond geometry (Å, º)

**Table:**

*D*—H···*A*	*D*—H	H···*A*	*D*···*A*	*D*—H···*A*
N1—H1*N*1···O2^i^	0.89 (2)	1.96 (2)	2.7772 (19)	152 (2)
N1—H2*N*1···O1^ii^	0.88 (2)	1.83 (2)	2.7047 (19)	174 (2)
N2—H1*N*2···O3^iii^	0.90 (3)	2.11 (3)	2.970 (3)	161 (3)
N2—H2*N*2···O3^ii^	0.84 (3)	2.34 (3)	3.092 (3)	149 (2)
C2—H2···O1^iv^	0.98	2.53	3.469 (2)	160

Symmetry codes: (i) −*x*+1, *y*−1/2, −*z*+3/2; (ii) *x*+1, *y*, *z*; (iii) *x*+1/2, −*y*+3/2, −*z*+2; (iv) −*x*+1, *y*+1/2, −*z*+3/2.
