# Supplementary figures and images for: Redetermined crystal structure of N-(β-carb­oxy­eth­yl)-α-isoleucine
Source: Acta Crystallogr E Crystallogr Commun. 2015 Aug 22;71(Pt 9):o665–6. doi: 10.1107/S2056989015014498 (PMC4555364; doi:10.1107/S2056989015014498)

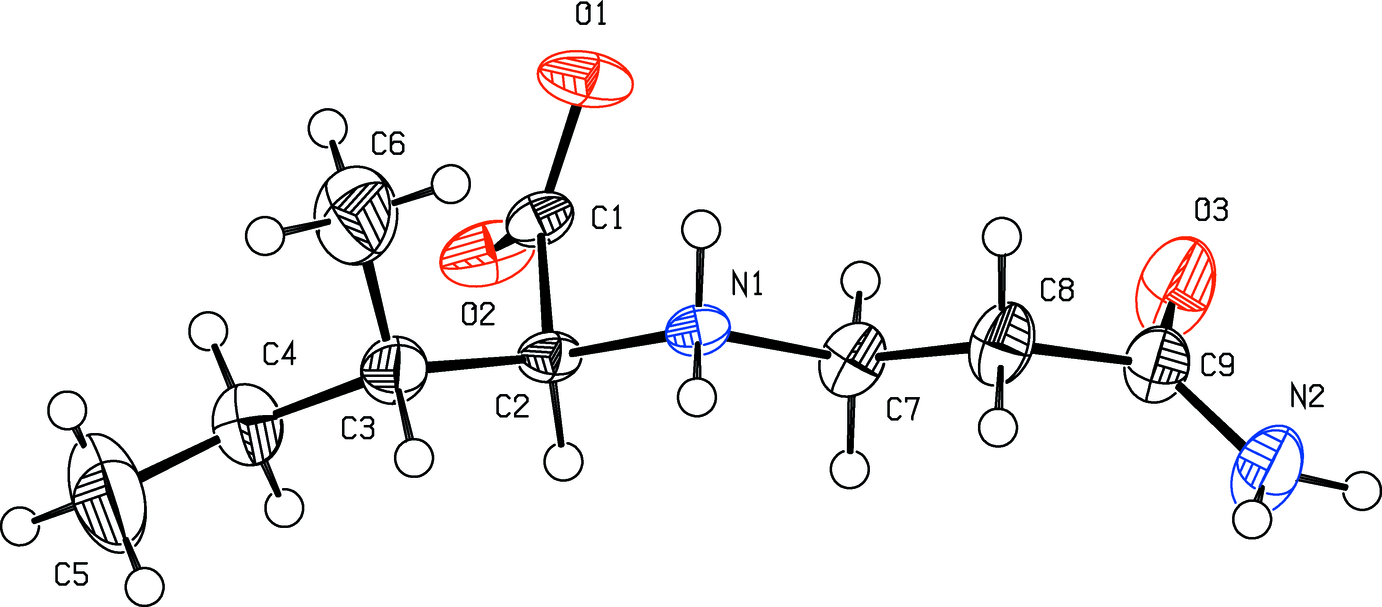

Supplement: Supplementary file 4 [file e-71-0o665-fig1.tif]

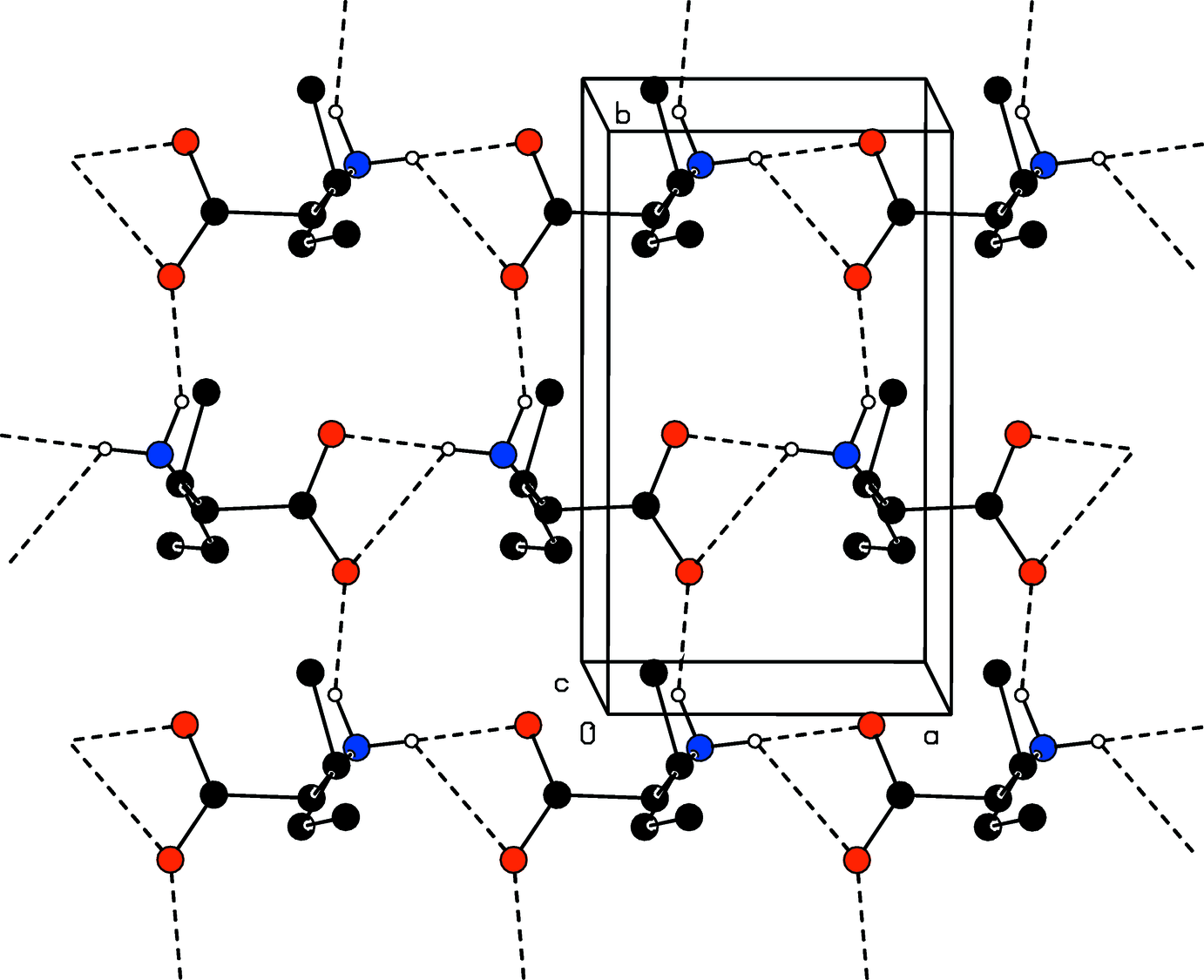

Supplement: Supplementary file 5 [file e-71-0o665-fig2.tif]

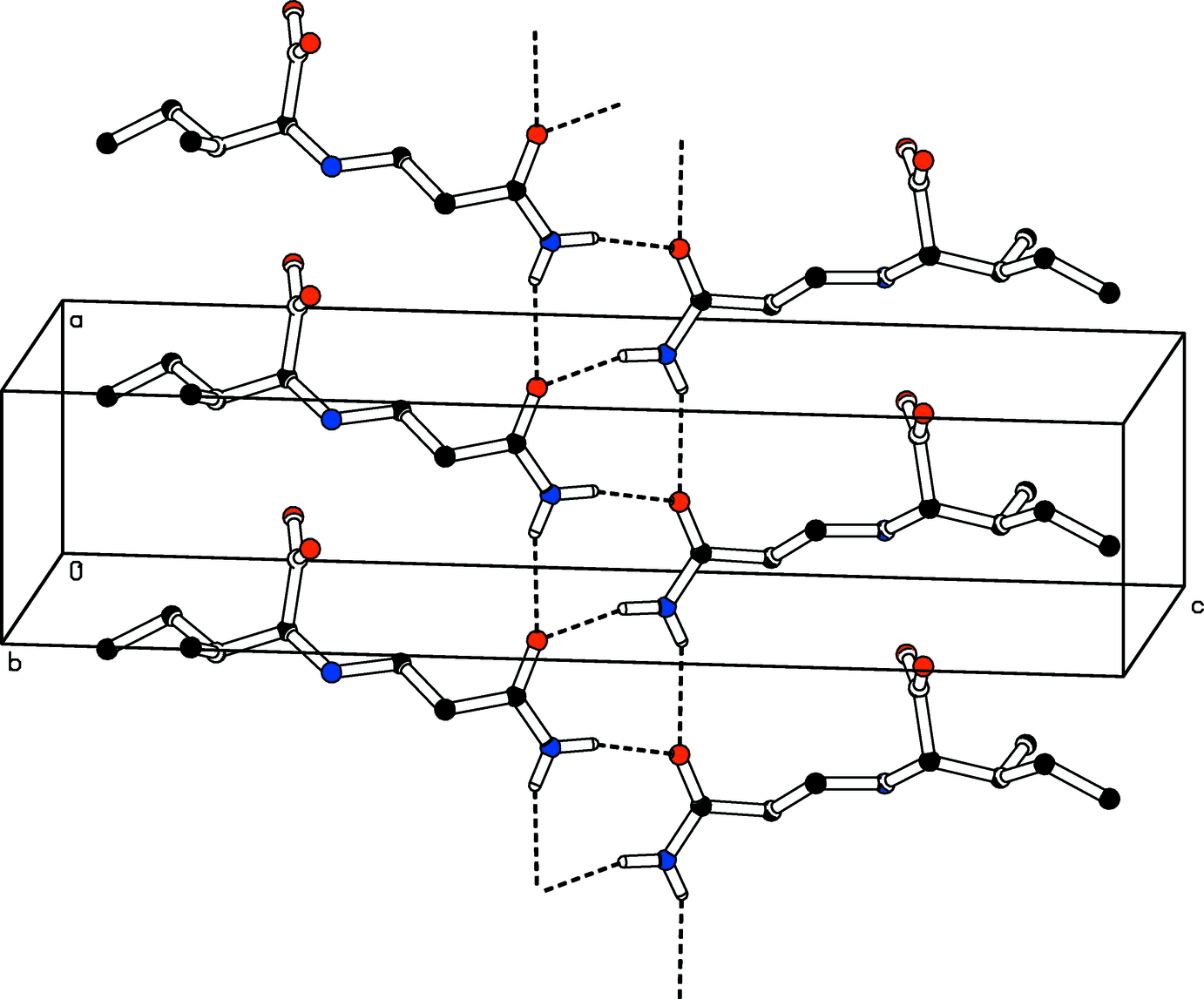

Supplement: Supplementary file 6 [file e-71-0o665-fig3.tif]
